# Vom Handwerker zum klinischen Pharmazeuten – Zur Geschichte der Apothekerausbildung in Deutschland

**DOI:** 10.1007/s00103-025-04037-1

**Published:** 2025-03-25

**Authors:** Axel Helmstädter

**Affiliations:** Institut für Geschichte der Pharmazie und Medizin, Philipps-Universität, Roter Graben 10, 35037 Marburg, Deutschland

**Keywords:** Apothekerausbildung, Pharmaziestudium, Geschichte, Klinische Pharmazie, Approbationsordnung, Pharmacist training, Pharmacy curriculum, History of pharmacy, Clinical pharmacy, Pharmacist licensing

## Abstract

Der Apothekerberuf mit ursprünglich handwerklichem Schwerpunkt wurde im Zuge der aufkommenden Naturwissenschaften, insbesondere der Chemie, seit dem 18. Jahrhundert zunehmend wissenschaftlich geprägt. Parallel wurden auch die Ausbildungsinhalte dergestalt angepasst, dass praktische Ausbildungszeiten reduziert und die universitäre Ausbildung verlängert wurde. Das ursprünglich (1875) nur 3‑semestrige Studium verlängerte sich auf 4 (1904), 6 (1934), 7 (1971) und letztlich auf 8 Semester (1989). In der Deutschen Demokratischen Republik verlief die Akademisierung dynamischer, dort wurde bereits 1951 8 Semester unterrichtet. Inhaltlich war die Aufwertung durch gestiegene berufliche Anforderungen in der pharmazeutischen Technologie, der Arzneimittelsynthese, der Pharmakologie und jüngst der klinischen Pharmazie notwendig geworden. Allerdings erfolgten die Anpassungen jeweils mit großer zeitlicher Verzögerung, die teilweise durch Eigeninitiative weitsichtiger Apotheker überbrückt wurden. Die jeweiligen Reformen blieben zudem gewöhnlich hinter den Forderungen und Erwartungen des Berufsstandes zurück, sodass sich die Diskussion um eine zeitgemäße Ausbildung meist unmittelbar nach Erlass einer neuen Prüfungsordnung fortsetzte.

## Abstract (en)

The profession of pharmacist, which originally focused on craftsmanship, became increasingly scientific in nature from the 18th century onwards as the natural sciences, particularly chemistry, emerged. In parallel, the content of training was adapted in such a way that practical training periods were reduced and university training was extended. The original three-semester course (1875) was extended to four (1904), six (1934), seven (1971), and finally to eight semesters (1989). In the German Democratic Republic, academization was more dynamic, with eight semesters already being taught there in 1951. In terms of content, the upgrading had become necessary due to increased professional requirements in pharmaceutical technology, drug synthesis, pharmacology and, most recently, clinical pharmacy. However, the adjustments were always made with a long delay, which was partly bridged by the initiative of far-sighted pharmacists. Moreover, the respective reforms usually fell short of the demands and expectations of the profession, so that the discussion about modern training usually continued immediately after new regulations had been issued.

## Einleitung

Wie der Schweizer Pharmaziehistoriker François Ledermann formuliert hat, ist „der Übergang des Apothekers vom Handwerker zum Akademiker überall in Europa im Laufe des 19. Jahrhunderts wohl das wichtigste Element der pharmazeutischen Vergangenheit“ [1]. Parallel hierzu ist auch ein Wandel der pharmazeutischen Ausbildung zu beobachten. Allerdings reagierten die Verordnungsgeber meist erst mit zeitlicher Verzögerung auf die veränderten beruflichen Anforderungen, statt programmatisch vorzugehen oder zukünftige und bereits absehbare Anforderungen des Berufes in den Blick zu nehmen. Eine jahrhundertelange handwerkliche Tradition wich einer fortschreitenden Akademisierung seit Anfang des 19. Jahrhunderts als Konsequenz einer zunehmenden Verwissenschaftlichung der Arzneimitteltherapie und -herstellung. Seither wird regelmäßig eine Anpassung von Ausbildungsinhalten an den pharmakotherapeutischen Fortschritt diskutiert und vorgenommen. Der historische Horizont und damit auch der Inhalt dieses Beitrages reichen von den handwerklichen Anfängen bis zur Diskussion um Ausbildungsinhalte im 21. Jahrhundert.

## Lehrling und Geselle

Die in der Pharmazie gängige Bezeichnung „Offizin“ für den Verkaufsraum einer Apotheke, der sich vom lateinischen „officina“ für „Werkstatt“ ableitet, weist noch heute auf die handwerkliche Tradition des Berufes hin. Hier wurde nicht in erster Linie verkauft und informiert, sondern es wurden die für Patienten individuell rezeptierten Arzneimittel manuell und mit einfachen technischen Hilfsmitteln zur unmittelbaren Anwendung hergestellt. Die Ausbildung des Nachwuchses folgte denn auch den Gebräuchen des Handwerks und unterschied sich prinzipiell nicht grundlegend von derjenigen der Schuster, Müller oder Schneider, wenn man von dem Erfordernis einer gewissen Kenntnis der lateinischen Sprache absieht, in der die Rezepte und Arzneibücher abgefasst waren.

Im Alter zwischen 14 und 16 Jahren begaben sich junge Männer in die Obhut eines Lehrherrn, der während der nächsten 4–6 Jahre für ihre rein praktische Ausbildung, aber auch für Kost und Logis sorgte. Hierfür war Lehrgeld zu entrichten. Gewöhnlich lebten die Lehrlinge im Haushalt ihres Prinzipals, der sich meist im gleichen Gebäude befand. Autobiografische Dokumente belegen eindrucksvoll das Sprichwort von den Lehr- und Herrenjahren [2], wobei die konkreten Arbeits- und Lebensumstände natürlich von den örtlichen Gegebenheiten und der Person des Prinzipals abhingen. Für längere Zeit umfassten die Tätigkeiten insbesondere die Vorbereitung und Reinigung von Arbeitsmitteln für die Rezeptur, später auch die Lagerung, Trocknung und Zerkleinerung der „Drogen“, also der pharmazeutisch verwendeten Pflanzen und Pflanzenteile. Der Arbeitstag konnte problemlos 15 h dauern, freie Tage gab es nur einige wenige im Jahr. Eine wie immer geartete theoretische Fundierung der Tätigkeiten fand nicht statt, Lehrbücher im eigentlichen Sinne gab es nicht, als literarische Quelle diente meist nur das jeweils gültige Arzneibuch, das jedoch kaum mehr als reine Arbeitsvorschriften enthielt.

**Abb. 1:**
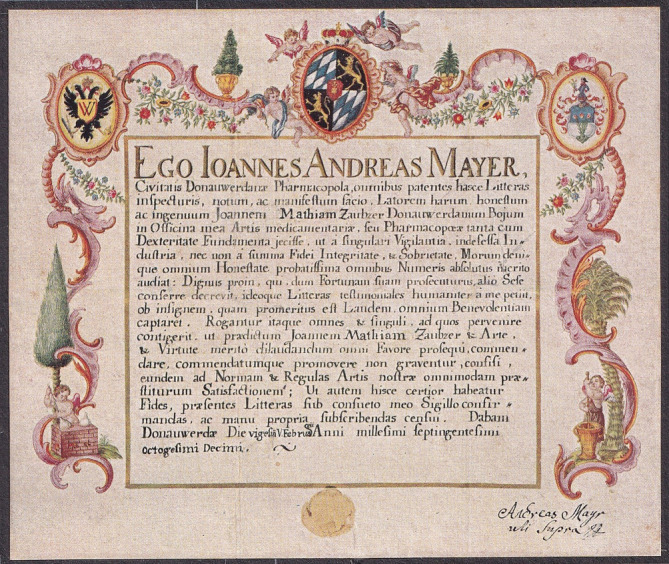
Lehrbrief für den späteren Apotheker Matthias Zaubzer (1780) aus [20]

Am Ende der Ausbildung erhielt der Lehrling ein Zeugnis bzw. einen Lehrbrief, der oft aufwendig gestaltet war (Abb. 1) und zur Erwerbsarbeit in einer Apotheke berechtigte. Es folgten meist, wie im Handwerk der Zeit üblich, „Wanderjahre“ mit Beschäftigung in verschiedenen Betrieben. Die Lebensumstände der „Apothekergehilfen“ genannten Gesellen blieben meist weiterhin prekär, unterschieden sich allerdings nur graduell von denjenigen anderer Handwerksgesellen [3]. Nach 2–4 Jahren konnte dann ein Examen vor einem Ärztekollegium abgelegt werden, bei dem unter anderem eine komplizierte Arzneizubereitung, quasi als „Meisterstück“, anzufertigen war. Es bildete den Abschluss der Ausbildung zum Apotheker und war Voraussetzung für eine Anstellung als sogenannter Provisor oder, falls das Kapital aufgebracht werden konnte, den Betrieb einer eigenen Apotheke.

## Ein erstes Ausbildungsdilemma

Im Grunde änderte sich an diesem Ablauf bis weit ins 19. Jahrhundert wenig; allerdings hatte sich schon seit dem Zeitalter der Aufklärung immer deutlicher gezeigt, dass auch Medizin und Pharmazie vom Aufkommen der Naturwissenschaften profitierten. Vor allem die Chemie erlebte seit dem letzten Drittel des 18. Jahrhunderts einen großen Aufschwung. Herausragenden Anteil hatten Apotheker, die sich für die neuen chemischen Erkenntnisse und Verfahren begeistern konnten, die sie sich aber durch Literaturstudium weitgehend autodidaktisch aneignen mussten; an Universitäten waren sie höchstens als Gasthörer geduldet. Allerdings waren sie laborchemisches Arbeiten aus der Apotheke gewohnt, deren apparative Ausstattung zum wissenschaftlichen Experimentieren genutzt werden konnte.

Tatsächlich bildeten Apotheker etwa zwischen 1750 und 1850 die Speerspitze des chemischen Fortschritts, etwa als Entdecker chemischer Elemente oder bei der Isolierung von Pflanzeninhaltsstoffen. Ein frühes wichtiges Beispiel ist die Entdeckung des Sauerstoffs durch den deutsch-schwedischen Apotheker Carl-Wilhelm Scheele (1742–1786), die zu einem kompletten Umbruch der theoretischen Chemie führen sollte. Immense wirtschaftliche Bedeutung gewann beispielsweise die 1747 gelungene Isolierung des pharmazeutischen Rohstoffs Zuckers aus der einheimischen Runkelrübe durch den Berliner Apotheker Andreas Sigismund Marggraf (1709–1782), die einen Import südamerikanischen Zuckerrohrs überflüssig machte. In pharmakotherapeutischer Hinsicht herausragend ist die Isolierung des Alkaloids Morphin durch den westfälischen Apotheker Friedrich Wilhelm Sertürner (1783–1841) im Jahr 1804 aus dem Opium, wodurch eine moderne und hochwirksame Therapie stärkster Schmerzen möglich wurde. Die überkommene Ausbildung hatte mit all dem allerdings nicht Schritt gehalten und verharrte noch immer im stupiden Erlernen manueller Operationen ohne systematisch-theoretische Fundierung, an der letztlich auch der Staat wenig Interesse zeigte. Ein zusätzliches formales Hindernis für eine Hochschulausbildung war sicherlich auch die Tatsache, dass die Personen, die den Apothekerberuf anstrebten, gewöhnlich kein Abitur nachweisen konnten.

Diesem „Ausbildungsdilemma“ versuchten engagierte Apotheker dadurch abzuhelfen, dass sie in Eigeninitiative eine vertiefte, mit einem Universitätsstudium nahezu vergleichbare Ausbildung in ihren Apotheken anboten. Das erste dieser „Privatinstitute“ [4] gründete 1779 Johann Christian Wiegleb (1732–1800) im thüringischen Langensalza. Das bedeutendste einer mittleren 2‑stelligen Zahl solcher Einrichtungen leitete Johann Bartholomäus Trommsdorff (1770–1832) in Erfurt mit seiner „Chemisch-physikalischen und pharmaceutischen Pensionsanstalt für Jünglinge“. Hier unterrichtete er in Vorlesungen und Laborpraktika etwa 300 Schüler, darunter Heinrich Emmanuel Merck (1794–1855), dessen Fähigkeit zur Reindarstellung von Alkaloiden die Grundlage für das bekannte pharmazeutische Unternehmen in Darmstadt legte. Trommsdorff und die anderen Leiter derartiger Ausbildungsstätten versuchten von Anfang an, staatliche Anerkennung oder Unterstützung zu erhalten, was weitgehend misslang. Erst 1823, also nach 28 Jahren erfolgreicher Lehrtätigkeit, erhielt Trommsdorff einen kleinen Zuschuss zu seiner Einrichtung und die Zusage für eine verkürzte Ausbildungszeit seiner Studenten. Die Notwendigkeit einer stärker wissenschaftlichen Bildung verspürten im Gegensatz dazu aber die Gehilfen selbst, die sich in Vereinen zusammenschlossen, um ihre fachliche Fortbildung zu betreiben, etwa die „Pharmazeutische Gesellschaft zu Berlin“, die 1796 auf Anregung eines Gehilfen gegründet worden war. Diese Vereinigungen organisierten Vorlesungen und Exkursionen oder richteten eine Bibliothek für ihre Mitglieder ein [3]. Sie dienten dem Erfahrungsaustausch, „der gegenseitigen Belehrung über wissenschaftliche Probleme der Apothekerkunst“ [5].

## Anfänge des obligatorischen Hochschulstudiums

Die Regierungen der deutschen Länder reagierten indes sehr zögerlich auf die Verwissenschaftlichung der Pharmazie; vergleichsweise fortschrittlich agierte das Königreich Bayern, dass 1808 eine 2‑jährige Hochschulausbildung verbindlich vorschrieb [6, S. 615–618, 7], wobei angehende Pharmaziestudenten von der Erfordernis des Abiturs befreit wurden (Tab. 1). Diese Situation blieb letztlich bis 1921 bestehen mit der Eigentümlichkeit, dass Pharmaziestudenten nicht „immatrikuliert“, sondern „inscribiert“ wurden und dass Hochschullehrer aus der reinen Apothekerlaufbahn vor ihrer Berufung erst noch das Reifezeugnis ablegen mussten. Anders als in Bayern konnte man sich in Preußen nicht dazu durchringen, ein Studium vorzuschreiben, sah aber durchaus die Notwendigkeit, den theoretischen Ausbildungsanteil zu erhöhen. Der Hochschulbesuch blieb prinzipiell freiwillig, Anreize dafür setzte der Staat aber in der Möglichkeit, von den in der Gehilfenzeit abzuleistenden 5 „Servierjahren“ bis zu 2 durch einen Universitätsbesuch zu ersetzen [6, S. 621]. Ebenso gingen auch andere deutsche Länder vor, etwa Baden und Württemberg.

**Tab. 1:** Übersicht zur Entwicklung der akademischen Apothekerausbildung in Deutschland (mod. n. [16, 21])

Jahr	Studienvoraussetzungen	Studiendauer	Famulatur	Nachuniversitäre praktische Ausbildung
*a) Deutsches Reich/Bundesrepublik Deutschland bis 1989/wiedervereinigtes Deutschland*				
1808 (Bayern)	Mindestens 3 Jahre Lehrzeit 2 Jahre Gehilfenzeit	2 Jahre	–	–
1864 (Preußen)	Höhere Schule 3 Jahre Lehrzeit 3 Jahre Gehilfenzeit	1,5 Jahre^a^	–	–
1875	Obersekundarreife 3 (mit Abitur 2) Jahre Lehrzeit 3 Jahre Gehilfenzeit	1,5 Jahre	–	–
1904	Primarreife 3 (mit Abitur 2) Jahre Lehrzeit 3 Jahre Gehilfenzeit	2 Jahre	–	2 Jahre^b^
1921	Abitur 2 Jahre Praktikum 1 Jahr Assistentenzeit	2 Jahre	–	2 Jahre^b^
1934	Abitur 2 Jahre Praktikum	3 Jahre	–	1 Jahr^b^
1971	Abitur^d^	3,5 Jahre	–	1 Jahr^c^
1989	Abitur	4 Jahre	2 Monate	1 Jahr^c^
2001	Abitur	4 Jahre	2 Monate	1 Jahr^c^
Aktuelle Forderung der Verbände	Abitur	5 Jahre	1 Monat	1 Jahr^c^
*b) Deutsche Demokratische Republik*				
1951	Abitur 1 Jahr Praktikantenzeit	4 Jahre	–	2 Jahre^b^
oder	Abitur	4 Jahre	3‑mal 6 Wochen	2 Jahre^b^
1955	Abitur	4 Jahre	1 Jahr	1 Jahr^b^
1968	Abitur	4,5 Jahre^e^	5 Monate	1 Jahr^b^
oder	Abitur	3,5 Jahre	5 Monate	2 Jahre^b^
1975	Abitur	5 Jahre^f^	3‑mal 1 Monat	1 Jahr^b^
1984	Abitur 1 Jahr Praktikum	5 Jahre^f^	–	1 Jahr^b^

^a^ Ersetzbar durch ein weiteres „Servierjahr“ pro Semester

^b^ „Kandidatenzeit“

^c^ „Pharmaziepraktikum“

^d^ Latinum nicht mehr verpflichtend

^e^ Einschließlich 6 Monate Diplomarbeit

^f^ Einschließlich 12 Monate Diplomarbeit

**Abb. 2:**
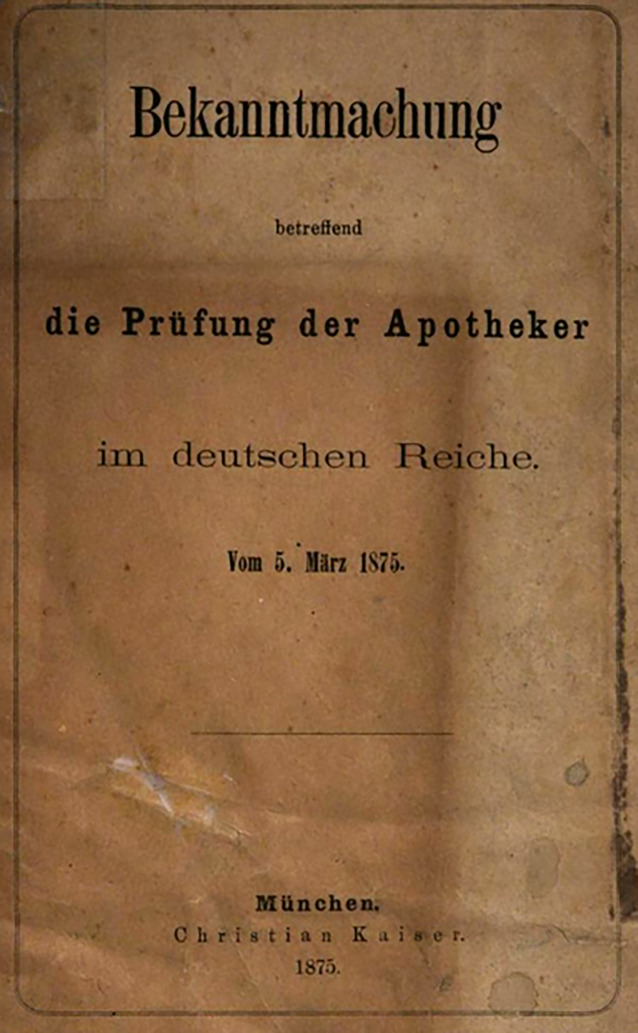
Bekanntmachung betreffend die Prüfung der Apotheker im deutschen Reiche vom 05.03.1875. Abbildung frei verfügbar unter [8]

Erst im Zuge der Gründung des Deutschen Reiches 1871 kam es dann zur ersten reichsweiten verpflichtenden Hochschulausbildung für Apotheker, die ein 3‑semestriges Studium in Verbindung mit je 3 Jahren Lehr- und Gehilfenzeit vorsah. Die Prüfungsordnung, die am 01.10.1875 in Kraft getreten war, sah durchaus anspruchsvolle botanische, chemische, physikalische und rechtliche Inhalte vor, deren Beherrschung in einem 5‑teiligen Examen in Theorie und Praxis nachzuweisen war (Abb. 2; [8]). Als Vorbildung war die Obersekundarreife ausreichend. Bereits unmittelbar danach setzte eine rege Diskussion im Berufsstand zu einer Reform dieser ersten Ausbildungsordnung ein: Schon 1879 setzte der Deutsche Apotheker-Verein eine Kommission ein, die Vorschläge erarbeiten sollte. Sie forderte das Abitur als Zugangsvoraussetzung, damals bereits ein Studium von 8 Semestern, ergänzt durch 3 Jahre „Servierzeit“. Bis zur Jahrhundertwende folgte eine anhaltende Diskussion verschiedener Varianten im Berufstand, der sich allerdings bei der Politik kein Gehör verschaffen konnte.

## Ausbildungsreformen bis zum Zweiten Weltkrieg

Anfang des 20. Jahrhunderts hatten allerdings wissenschaftliche Entwicklungen, insbesondere auf dem Gebiet der Hygiene, eine Reform unausweichlich gemacht. Sie folgte mit der Prüfungsordnung vom 18.05.1904. Diese ließ einen höheren wissenschaftlichen Standard indes nur in Ansätzen erkennen, denn die berufspraktischen Ausbildungsinhalte überwogen weiterhin und das Studium wurde lediglich um ein Semester verlängert, während der Berufsstand 6–8 Halbjahre gefordert hatte. Als Zugangsvoraussetzung wurde noch immer kein Abitur, sondern nur die sogenannten Primarreife verlangt. Abzuleisten waren eine 3‑jährige (mit Abitur 2‑jährige) Lehrzeit, abzuschließen mit einer praktischen Prüfung, ein weiteres Jahr als „Gehilfe“ in einer Apotheke, dann das 4‑semestrige Studium und anschließend nochmals 2 Jahre in der Praxis. Die Ausbildungszeit betrug also insgesamt 8 (bzw. 7) Jahre, von denen nur ein Viertel auf das Studium entfiel.

Das für den Deutschen Apotheker-Verein (DAV) unbefriedigende Ergebnis führte zu einer sich unmittelbar fortsetzenden Diskussion, die 1920 einen kleinen Erfolg verbuchen konnte: Mit Wirkung vom 01.01.1921 wurde das Abitur zur Eingangsvoraussetzung für das Pharmaziestudium. Eine erneut geforderte Verlängerung des Studiums auf 6 Semester lehnte die Regierung ab, die Diskussion darüber im Berufsstand setze sich indes unvermindert fort. Schon damals wurde darauf verwiesen, dass das deutsche Pharmaziestudium das im europäischen Vergleich kürzeste sei, was dem Ansehen der deutschen Apotheker schade [9]. Es wurde zudem insbesondere angesichts des 1926 neu herausgebrachten Arzneibuchs (DAB 6) deutlich, dass 4 Semester nicht ausreichten, um die notwendigen wissenschaftlichen Grundlagen für den Apothekerberuf zu legen. Insbesondere die geforderten Sterilisationsmethoden waren Neuland, sie katalysierten letztlich auch die Entwicklung der pharmazeutischen Technologie als Hochschuldisziplin [10].

Erneut dauerte es aber Jahre bis zur Einführung entsprechenden Pflichtunterrichts in der Prüfungsordnung, die, im Dezember 1934 beschlossen, am 01.04.1935 in Kraft trat. Sie sah eine 2‑jährige Lehrzeit am Beginn der Ausbildung vor, dann endlich 6 Semester Studium sowie ein einjähriges „Kandidatenjahr“ zum Abschluss. An neuen und zeitgemäßen Inhalten umfasste die universitäre Lehre nicht nur Grundzüge der Bakteriologie, Hygiene und Sterilisationsverfahren, auch physiologisch-chemische Untersuchungen, die zum damaligen pharmazeutischen Berufsbild gehörten; auch pharmakologische Lehrinhalte gab es schon, ebenso Kurse in Homöopathie und Wirtschaftslehre. Nicht alles war allerdings wissenschaftlich begründet, manche Lehrinhalte verdankten ihre Existenz dem nationalsozialistischen Weltbild, das etwa der Homöopathie als „typisch deutscher“ Heilmethode sehr offen gegenüberstand, oder den Autarkiebemühungen des „Dritten Reiches“, die eine Rückbesinnung auf einheimische Arzneipflanzen und eine Wiederbelebung der Eigenherstellung in Apotheken begründeten [11].

## Ausbildung nach dem Zweiten Weltkrieg in der Bundesrepublik

Nach Ende des Krieges konnte die Apothekerausbildung nur schleppend wieder aufgenommen werden. Der Wiederaufbau einer funktionierenden Arzneimittelversorgung aus Ruinen drängte Fragen der Ausbildungsinhalte naturgemäß in den Hintergrund, obwohl bereits 1949 umfassende Reformvorschläge aus Kreisen der Hochschullehrer gemacht worden waren. Sie wurden vom Vorsitzenden der im Januar 1949 gegründeten „Vereinigung der Direktoren der pharmazeutischen Hochschul-Institute“, Hans-Paul Kaufmann (1889–1971), vorgelegt, der jedoch klar sah, dass keine unmittelbare Realisierungsmöglichkeit bestand. Er forderte angesichts der Vielzahl inzwischen verfügbarer synthetischer Arzneistoffe unter anderem Unterricht in Arzneistoffsynthese.

Bereits unmittelbar nach dem Krieg forderten auch andere Gremien eine möglichst schnelle Weiterentwicklung der noch gültigen Ausbildungsordnung von 1934, so auch die „Arbeitsgemeinschaft Pharmaziestudenten“, die zunächst 7, dann 8 Semester mit medizinischen Inhalten und Pharmakologie als Prüfungsfach vorschlug. In der Folge verging auch kaum ein Apothekertag, der sich nicht mit Ausbildungsfragen befasst hätte. Hier und in diversen Kommissionen und Arbeitsgruppen diskutierte man zahlreiche Modelle im Detail, die praktisch alle eine Verlängerung des Studiums auf 8 Semester vorsahen. Großen Raum nahm die Frage nach Umfang und Zeitpunkt der praktischen Ausbildungszeiten in Anspruch. Neben einer Positionierung vor und nach dem Studium wurde auch diskutiert, ein praktisches Jahr zwischen Grund- und Hauptstudium einzuschieben.

Von politischer Seite sah man die Notwendigkeit einer Reform auch, hielt es aber für sinnvoll, zunächst Grundsatzfragen des Apothekenwesens anzugehen, so ein 1960 erlassenes Apothekengesetz und, als Grundlage einer reformierten Ausbildung, eine Bundesapothekerordnung, die allerdings erst 1968 in Kraft trat, sodass Ausbildungsfragen noch Jahre aufgeschoben wurden. In die anhaltende Diskussion war auch der Wissenschaftsrat eingetreten, der 1964 vorschlug, die Lehrzeit entfallen zu lassen und 6 Semester Studiendauer beizubehalten. Angesichts der unbestritten großen Stofffülle wurde angeregt, verstärkt Kurse in die vorlesungsfreie Zeit zu verlegen, um die Ausbildungszeit nicht verlängern zu müssen. Hier findet sich auch der Vorschlag einer zweigleisigen Ausbildung: Eine nachuniversitäre einjährige „Kandidatenzeit“ sollte direkt zur Approbation führen, ein leistungsstarken Absolventen vorbehaltenes Aufbaustudium mit Abschlussarbeit für Aufgaben in anderen Bereichen wie Industrie und Wissenschaft qualifizieren. Eine Approbation wäre in diesem Modell mit einer auf 6 Monate verkürzten Kandidatenzeit anschließend noch möglich gewesen. Die Vorschläge des Wissenschaftsrates wurden allerdings von allen Gremien der Pharmazie abgelehnt [12].

Der Apothekerstand blieb, wiederum mit dem Argument einer im internationalen Vergleich minderwertigen Ausbildung, bei seiner Forderung nach 8 Semestern und lehnte auch einen Diplomstudiengang ab, da er zu einer Abwertung des nichtdiplomierten Apothekerberufs führe. Allerdings hatte die Universität Kiel bereits 1951 eine Diplomprüfungsordnung erlassen. Ein solcher Studiengang, den auch der einflussreiche Marburger Hochschullehrer Horst Boehme (1908–1996) befürwortete, blieb, gegen alle Widerstände, in den 1960er-Jahren Anliegen der Hochschullehrer für den Fall, dass eine Verlängerung des Studiums auf 8 Semester scheitern sollte. Letztlich ließ man davon ab; nur die Universität Marburg bot in Westdeutschland einen Diplomstudiengang an, den allerdings nur eine Absolventin tatsächlich abschloss. Die Diskussion fand schließlich ihr Ende mit dem Erlass der Bundesapothekerordnung von 1968, die eine Mindestausbildungsdauer von 4,5 Jahren einschließlich 12 Monate praktischer Ausbildung festlegte [7, S. 131, 13].

Den Gremien blieben dann noch 3 Jahre Zeit, eine genaue Approbationsordnung auszuarbeiten, die zum Wintersemester 1971/1972 wirksam wurde. Sie sah einen Wegfall des Vorpraktikums, eine Studiendauer von 7 Semestern mit den neuen Prüfungsfächern Pharmakologie und pharmazeutische Technologie und ein anschließendes praktisches Jahr als einzigen pharmazeutischen Ausbildungsgang vor. Dies hatte mittelbar auch Konsequenzen bezüglich weiterer pharmazeutischer Berufe, denn es entfiel der „Vorexaminierte“ als Arbeitskraft. So nannte man Apothekenmitarbeiter, die nicht studiert, aber nach dem 2‑jährigen Vorpraktikum ein Examen abgelegt hatten. Den dadurch zu befürchtenden Arbeitskräftemangel in Apotheken kompensierte man durch die Einführung des Berufsbildes „Pharmazeutisch-technischer Assistent“ (PTA; [14]), der pharmazeutische Tätigkeiten und Aufsicht eines Apothekers ausführen darf.

## Ausbildung nach dem Zweiten Weltkrieg in der DDR

Die akademische Aufwertung der pharmazeutischen Ausbildung gelang in der DDR tatsächlich ungleich schneller als im Westen, so war bereits 1951 das Studium auf 8 Semester ausgedehnt worden. Pharmazeutische Technologie und Pharmakologie wurden vertieft und zu Prüfungsfächern aufgewertet; hinzu trat systemtypisch obligatorischer Unterricht in Politik und der russischen Sprache. Unsicher war man offensichtlich bezüglich des praktischen Ausbildungsanteils; das 1951 zugunsten eines zweiten praktischen Jahres abgeschaffte Vorpraktikum wurde 1955 wieder vorgezogen, 1968 mit der folgenden Reform von 1968 dann endgültig gestrichen. Diese Neuordnung sah dann aber eine Diplomarbeit vor, für die zunächst 6, später 12 Monate eingeplant waren. 1975 wurde der Diplomstudiengang auf 10 Semester erweitert, wobei zu berücksichtigen ist, dass im Sozialismus für unabdingbar gehaltene politisch-gesellschaftliche Fächer auch ihren Platz beanspruchten.

Allerdings übernahm man moderne Lehrinhalte viel unmittelbarer als im Westen. So gab es bereits ab 1968 „Datenverarbeitung“; auch das von den ostdeutschen Hochschullehrern Siegfried Pfeifer (1926–2021), Peter Pflegel (1939–2017) und Hans-Hubert Borchert (1944–2014) maßgeblich entwickelte Fach „Biopharmazie“ [15] wurde alsbald Prüfungsgegenstand, während es im Westen nur in gewissem Umfang in den pharmazeutisch-technologischen Unterricht integriert wurde. Wahrscheinlich „tat man sich in unter dirigistischen und totalitären Regimen durchaus leichter; … Vorstellungen der Entscheidungsträger … waren auf dem Wege von Erlassen sehr viel rascher durchzusetzen, zumal sie keinen Widerspruch erlaubten“ [16].

## Ausbildung nach der Wiedervereinigung – Klinische Pharmazie

In der Bundesrepublik gelang es, und zwar katalysiert von außen, erst kurz vor der Wiedervereinigung die Studienzeit auf 8 Semester zu verlängern. Schon immer war beklagt worden, dass die deutsche Pharmazeutenausbildung einem europäischen Vergleich qualitativ und quantitativ nicht standhielt. Nun drohte mit der 1987 erlassenen EU-Richtline zur gegenseitigen Anerkennung der Diplome die Arbeitsmöglichkeit deutscher Apotheker im EU-Ausland zu entfallen. Ohne wesentliche inhaltliche Diskussion wurde eiligst eine Ausweitung auf 8 Semester beschlossen, die bereits über 100 Jahre zuvor erstmals gefordert worden war. Inhaltlich beließ man es bei Marginalien wie der Einführung bestimmter Seminare oder der erstmals eröffneten Möglichkeit, weibliche Absolventinnen auf der Approbationsurkunde als „Apothekerin“ zu bezeichnen. Inhaltlich wurde gar gekürzt, weil die 8‑semestrige Ausbildung kostenneutral umgesetzt werden sollte. Diese Approbationsordnung vom 1989 wurde dann auch in das wiedervereinigte Deutschland übernommen, was aus ostdeutscher Perspektive zweifellos einen Rückschritt bedeutete.

Inhaltlichen Reformbedarf hätte es vor allem hinsichtlich der „klinischen Pharmazie“ gegeben, ein bereits in den 1960er-Jahren in den USA aufgekommenes Konzept, pharmazeutische Expertise zur Verbesserung der Arzneimitteltherapiesicherheit zu nutzen [17, 18]. „Klinisch“ als Übersetzung des englischen „clinical“, das eher „patientenorientiert“ bedeutet, darf man nicht auf die Situation im Krankenhaus beschränken. Damit verschob sich der Fokus des Apothekerberufs von Herstellung und Qualitätskontrolle der Arzneimittel hin zu deren sicherer Anwendung am und durch den Patienten. Entsprechend wurden, in den USA bereits in den frühen 1970er-Jahren, Vorschläge einer aus Hochschullehrern, Apothekern, Ärzten und einer Krankenschwester bestehenden Kommission umgesetzt, laborchemische Studieninhalte weitgehend durch medizinisch-pharmakologische zu ersetzen. Ähnlich agierte Großbritannien etwa 10 Jahre später auf Anregung des Gesundheitsministers, dessen politische Überzeugung es war, durch patientenorientierte pharmazeutische Expertise die medikamentöse Therapie verbessern zu können [19].

In Deutschland bahnte sich die klinische Pharmazie ihren Weg dagegen im Wesentlichen durch die Eigeninitiative süddeutscher Krankenhausapotheker, die sich auf eigene Kosten und in ihrer Freizeit entsprechende Kenntnisse aneigneten. Eine bedeutende Pionierleistung ist die Einführung der speziellen „Fachausbildung“ in klinischer Pharmazie durch den Bundesverband Deutscher Krankenhausapotheker (ADKA) im Jahr 1985. Dies geschah im Vorgriff auf die zu erwartenden, aber noch in der Beratung feststeckenden Weiterbildungsordnungen der Apothekerkammern der Länder, mit denen den Apothekern die postgraduale Qualifikation zum Fachapotheker eröffnet werden sollte. Dies geschah aber auch in dem Bewusstsein, dass gerade hinsichtlich klinischer Pharmazie die evidente Lücke zwischen Studieninhalten und modernen beruflichen Anforderungen geschlossen werden musste [20].

Einen ersten Lehrauftrag an einer deutschen Hochschule erhielt der im Städtischen Klinikum Karlsruhe tätige Pionier klinisch-pharmazeutischer Dienstleistungen Hans Joachim Meyer (1939–1997) im Jahr 1981 in Freiburg, 1986 wurde Werner Fürtig zum außerplanmäßigen Professor für klinische Pharmazie an der Universität Rostock ernannt. Eine Habilitationsmöglichkeit wurde im selben Jahr auf Initiative des pharmazeutischen Chemikers Walter Schunack (1935–2011) in Berlin eröffnet, die Ulrich Jaehde wahrnahm. Er wurde später (1999) auf die erste deutsche Professur Klinische Pharmazie an der Universität Bonn berufen. Noch immer war die Erteilung klinisch-pharmazeutischen Unterrichtes aber in das Belieben der einzelnen Hochschulen gestellt. Dies änderte sich erst mit einer erneuten Ausbildungsreform 2001, die klinische Pharmazie als Ausbildungs- und Prüfungsfach vorsah. Allerdings behielten, anders als in angelsächsischen Ländern und politisch gewollt, rein naturwissenschaftliche Inhalte ein deutliches Übergewicht. Dadurch und durch die in der Diskussion unbestrittene Beibehaltung des Staatsexamensstudiengangs konnte die Einheit der pharmazeutischen Ausbildung für alle Berufsfeder erhalten werden. Die Universitäten Freiburg und München etablierten daraufhin in eigener akademischer Machtvollkommenheit zusätzlich einen nicht zu Approbation führenden Bachelor‑/Masterstudiengang „Pharmazeutische Wissenschaften“. Einige Universitäten bieten zudem Masterstudiengänge an, die sich an das pharmazeutische Staatsexamen anschließen.

**Abb. 3:**
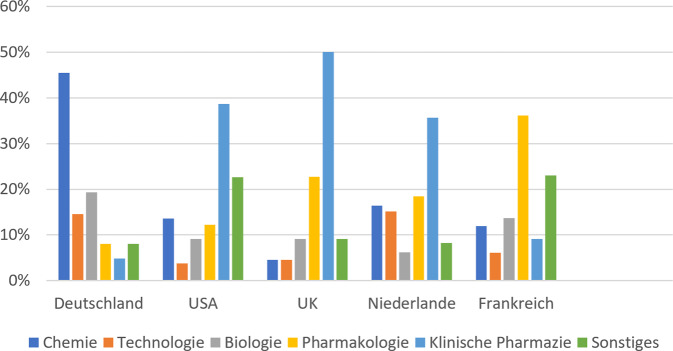
Ausbildungsinhalte nach Fächern in ausgewählten Ländern (eigene Abbildung mod. n. [23])

Der im internationalen Vergleich noch immer geringe klinische Anteil in der Approbationsordnung von 2001 (Abb. 3), aber auch der rasante Fortschritt beispielsweise hinsichtlich immunologischer und gentherapeutischer Heilverfahren führten indes schon nach kurzer Zeit zum erneuten Aufflackern der Diskussion um zeitgemäße Ausbildungsinhalte, die bis heute anhält. Mit Initiativen wie dem „kompetenzorientierten Lernzielkatalog Pharmazie“ der Bundesapothekerkammer (2017) sowie insbesondere dem 2022 verabschiedeten und von zahlreichen Verbänden konsentierten „Positionspapier Runder Tisch Novellierung der Approbationsordnung für Apotheker“ liegen fundierte und konkrete Vorschläge zu einer zeitgemäßen Ausbildung von Apothekerinnen und Apothekern vor.

## Fazit

Wesentliche Elemente in der Geschichte der Apothekerausbildung sind eine historisch starke handwerkliche Tradition sowie eine seit dem frühen 19. Jahrhundert zunehmende Akademisierung der Ausbildung, die sich an einer steten Abnahme berufspraktischer Lehrzeiten zugunsten des Universitätsstudiums zeigt. Sie blieb dennoch bis in jüngste Zeit hinter den akademischen Ausbildungszeiten anderer Industrienationen zurück. Die Ausbildung wandelte sich parallel zur zunehmenden Verwissenschaftlichung der Arzneimitteltherapie, -herstellung und -anwendung sowie zu neu aufkommenden beruflichen Erfordernissen und Aufgabengebieten. Allerdings ist festzuhalten, dass die Verordnungsgeber stets sehr zögerlich agierten und die Ausbildung nur langsam, unter äußerem Druck, beispielsweise durch EU-Vorgaben, und niemals in der Weise reformierten, wie es der Berufsstand gefordert hatte. Eine historische Rückschau erweckt gar den Eindruck, die Politik habe der Ausbildung der Personen, denen sie die „ordnungsgemäße Versorgung der Bevölkerung mit Arzneimitteln“ anvertraut, selten hohe Priorität eingeräumt. Mehrfach in der Geschichte, angefangen bei den „Privatinstituten“, zuletzt aber noch am Beispiel der „klinischen Pharmazie“, war es der Fall, dass offensichtlich notwendige neue Lehrinhalte zunächst und für längere Zeit in Eigenregie des Berufsstandes vermittelt werden mussten. Deren letztendliche universitäre Etablierung blieb meist unbefriedigend, sodass sich die berufspolitische Diskussion nach Erlass einer Ausbildungsordnung nahezu unmittelbar fortsetzte. Fragen der hochschulpolitischen Gesamtsituation, die einen Trend zu möglichst kurzer Studiendauer setzen wollte, sowie damit verbunden der Finanzierbarkeit wiesen die programmatischen Vorstellungen des pharmazeutischen Berufsstandes regelmäßig in die Schranken. So konstatiert die historische Forschung: „Den jeweiligen Reformen standen überdies meist finanziell bedingte Einschränkungen im Wege, wodurch zwingend notwendige Verbesserungen teils verzögert oder gar vereitelt, teils lediglich in einem Kompromiss verwirklicht wurden“ [7, S. 207].
